# An exploratory analysis of the impact of the COVID-19 pandemic on pediatric type 1 diabetes mellitus patient outcomes: A single-center study

**DOI:** 10.3389/fped.2022.1038345

**Published:** 2022-11-18

**Authors:** Raeesha Rajan, Uma Athale, Joycelyne Efua Ewusie, Karen McAssey, Lehana Thabane, M. Constantine Samaan

**Affiliations:** ^1^ Department of Pediatrics, McMaster University, Hamilton, ON, Canada; ^2^ Division of Pediatric Endocrinology, McMaster Children’s Hospital, Hamilton, ON, Canada; ^3^ Department of Health Research Methods, Evidence and Impact, McMaster University, Hamilton, ON, Canada; ^4^ Division of Hematology Oncology, McMaster Children’s Hospital, Hamilton, ON, Canada; ^5^ The Research Institute Biostatistics Unit, St Joseph’s Healthcare Hamilton, Hamilton, ON, Canada; ^6^ Department of Anesthesia, McMaster University, Hamilton, ON, Canada; ^7^ Centre for Evaluation of Medicines, Hamilton, ON, Canada

**Keywords:** COVID-19, type 1 diabetes mellitus, child, diabetic ketoacidosis, hypoglycemia, glycemic control, HbA1c

## Abstract

**Background:**

The COVID-19 pandemic led to substantial shifts in pediatric diabetes care delivery to virtual and hybrid models. It is unclear if these changes in care delivery impacted short-term patient outcomes.

**Objectives:**

We aimed to explore glycemic control and other diabetes-related outcomes in children living with Type 1 Diabetes Mellitus (T1DM) during the first year of the COVID-19 pandemic at a tertiary pediatric academic center in Canada.

**Subjects:**

Patients <18 years of age with a confirmed diagnosis of T1DM for at least one year were included.

**Methods:**

This was a retrospective chart review. We compared data from two years pre-pandemic (March 15, 2018–March 14, 2020) to the first year of the pandemic (March 15, 2020–March 14, 2021). The data assessed included glycemic control [Hemoglobin A1c (HbA1c)], diabetic ketoacidosis (DKA), hospital attendance and hospitalizations, hypoglycemia, and hyperglycemia. The generalized estimating equation (GEE) analysis was used to model potential factors affecting the HbA1c and diabetes-related morbidities. Multiple imputations were conducted as a sensitivity analysis.

**Results:**

There were 346 eligible patients included in the study. The HbA1c remained stable during the pandemic compared to the pre-pandemic phase (MD-0.14, 95% CI, −0.28, 0.01; *p* = 0.058). The pandemic saw an increase in the number of newly diagnosed patients (X^2 ^= 16.52, *p* < 0.001) and a higher number of newly diagnosed patients presenting in DKA (X^2 ^= 12.94, *p* < 0.001). In patients with established diabetes, there was an increase in hyperglycemia (OR1.38, 95% CI, 1.12,1.71; *p* = 0.003) and reduced DKA (OR 0.30, 95% CI, 0.12,0.73; *p* = 0.009) during the pandemic compared to the pre-pandemic phase. Stable rates of hospitalization (OR0.57, 95% CI, 0.31,1.04, *p* = 0.068) and hypoglycemia (OR1.11, 95% CI, 0.83,1.49; *p* = 0.484) were noted. These results were retained in the sensitivity analysis.

**Conclusions:**

Glycemic control in children with T1DM remained stable during the first year of the pandemic. There were more newly diagnosed patients during the pandemic compared to the pre-pandemic phase, and more of these new patients presented in DKA. The latter presentation was reduced in those with established diabetes during the same period.

Further studies are needed to assess the ongoing impact of the COVID-19 pandemic on T1DM care pathways and outcomes to allow children, families, and diabetes teams to personalize choices of care models.

## Introduction

In March 2020, the World Health Organization declared COVID-19 a global pandemic (1, 2). The then-novel SARS-CoV-2, the virus responsible for the pandemic, led to global lockdowns and social distancing measures to curb viral spread and to help manage finite healthcare resources (3–5). For most children, in-person schooling and organized activities were cancelled or moved to virtual platforms (6, 7). The resurgent waves of the virus variants continue to disrupt access to healthcare services, care delivery, schooling, social activities, work, and global economic activities (8).

During the pandemic, pediatric outpatient services for chronic conditions, including Type 1 diabetes mellitus (T1DM), pivoted to virtual care models including video and telephone consultations (9–11). Even with the up-scaling of vaccinations and relaxation of social distancing measures, in-person clinical services continued to be disrupted, and hybrid models of care have become commonplace (10, 12).

In-person visits were the norm for providing diabetes care pre-pandemic (13–15), and virtual consultations occurred infrequently (16, 17). The rapid adoption of virtual care demonstrated resilience in care delivery to support patients to maintain health outcomes during the pandemic (18). The effect of the pandemic on health outcomes, diabetes technology use, and care delivery patterns in children with T1DM is only emerging, and it will take time before the impact of the pandemic is fully evaluated, but the pre-pandemic limited evidence for the value of virtual care in T1DM was promising (18–26).

Emerging evidence suggests that there may be a rise in the number of children newly diagnosed with T1DM during the pandemic compared to the pre-pandemic phase, although this association has not been universal (27–33). The data so far also suggest an increase in the number of children presenting in diabetic ketoacidosis (DKA) (26, 34–36).

This study aimed to compare T1DM outcomes during the pandemic to the pre-pandemic phase. We tested the hypothesis that in children with T1DM, glycemic control and diabetes-related morbidities will worsen during the COVID-19 pandemic compared to the pre-pandemic phase.

## Methods

This study was a retrospective chart review that utilized data from the study of COVID-19 Effects on Glycemic Control in Children Living with Diabetes (CGC study).

We included boys and girls aged 2- < 18 years who attended the Pediatric Diabetes Program at McMaster Children's Hospital, a tertiary pediatric academic center in Ontario, Canada.

The study included patients who were diagnosed for at least one year with T1DM by the end of 2018 and had longitudinal follow-up data. The T1DM diagnosis ascertainment was based on standard criteria (13, 14). We excluded patients with cystic fibrosis-related diabetes, monogenic diabetes, medication-induced hyperglycemia, and type 2 diabetes. Patients less than 2 years of age were also excluded to avoid the potential inclusion of neonatal and genetic forms of diabetes.

We collected data for the two years pre-pandemic (March 15, 2018–March 14, 2020), and for the first year of the pandemic (March 15, 2020–March 14, 2021). At our center, clinical care was almost completely virtual from mid-March to September 2020. Then, a limited hybrid care delivery model was enacted with some in-person visits from October to December 2020. From January to March 2021, care returned to virtual platforms due to a new wave of the virus. The data from the pandemic phase, especially the Hemoglobin A1c (HbA1c), were limited by restrictions of patient access to local laboratory and clinic-based point-of-care testing. We included patients who had at least one HbA1c during the first year of the pandemic. We collected data including the number of newly diagnosed T1DM patients in the first year of the pandemic, age at diagnosis, diabetes duration, sex, visit date, treatment details including Multiple Daily Injections (MDI) or insulin pump therapy data, continuous glucose monitor (CGM) use, DKA, diabetes-related hospital attendance and hospitalizations, hypoglycemic events, and hyperglycemia.

We also collected available anthropometric data including weight, weight percentile, height, height percentile, and Body Mass Index (BMI) z-score (37). Of note, only a subgroup of patients had hospital-based height measured during the pandemic (*n* = 110, 31.80%), and weights included home-reported (*n* = 189, 61.00%) and hospital-measured (*n* = 122, 39.00%) data. We only included the BMI z-score data from the subset of patients who had weights and heights measured at the hospital to provide a conservative yet potentially more accurate data.

The main outcome of the study was the comparison of HbA1c change during the pandemic when compared to the pre-pandemic phase. Other outcomes included comparisons of the number of newly diagnosed T1DM cases, DKA, hospital attendance and hospitalizations, and any hypoglycemia and hyperglycemia at diagnosis and in those with established diabetes pre- and during the pandemic. Data on hypoglycemia and hyperglycemia were either reported or documented *via* CGM, glucometer, or logbook reviews.

DKA was defined as hyperglycaemia with blood glucose >11.00 mmol/L (200 mg/dL), venous blood gas-based pH <7.30, serum bicarbonate <15 mmol/L and the presence of ketones (ß-hydroxybutyrate ≥3 mmol/L in blood, moderate-large ketonuria) (39). Hospital attendance and hospitalizations refers to diabetes-related healthcare facility including emergency department visits or overnight hospital admissions. Hypoglycemia was defined as a plasma glucose level ≤3.9 mmol/L (70 mg/dL) and hyperglycemia was defined as a plasma glucose level ≥13.3 mmol/L (240 mg/dL) (39).

The Hamilton Integrated Research Ethics Board approved the study and granted a waiver of consent due to the anonymous and aggregate nature of the data used in the analyses. The study was performed in accordance with the guidelines of the Tri-Council Policy Statement 2 and the basic principles of Good Clinical Practice.

### Statistical analysis

Continuous variables are presented as means (SD) with range where indicated, and dichotomous variables are reported as numbers (%). Tests for collinearity were conducted to ensure predictors used in the model were not highly correlated. A variance inflation factor <5 was used to meet this assumption of non-collinearity (40).

We utilized the paired *t*-test for comparisons of continuous variables and McNemar's test for binary variables to report differences in repeated measures pre-pandemic when compared to the pandemic phase. To assess differences in the proportions of patients diagnosed pre-pandemic to those diagnosed during the pandemic and their clinical presentations, we utilized the Wald *χ*^2^ test. To assess the effect of time as a confounding variable on our measures of interest, we also utilized these tests to assess the differences in the pre-pandemic data from 2018 when compared to data from 2019.

The generalized estimating equation (GEE) model was applied to compare HbA1c levels before and during the pandemic and was adjusted for age, sex, treatment type, sensor use, weight and diabetes-related morbidities (41, 42). GEE models were also used to assess changes in hypoglycemia, hyperglycemia, DKA, and hospital attendance and hospitalizations (41). These models were adjusted for age, sex, treatment type, and sensor use (41, 43). For this analysis, we utilized the first-order auto-regressive working correlation matrix that recognizes correlations are highest between adjacent times and systematically decrease with increasing distance between time points (41, 43).

Continuous variables were reported using mean difference (MD) with a 95% confidence interval (CI) and binary variables were presented as an odds ratio (OR) with a 95% CI.

To assess the robustness of the findings, we employed a sensitivity analysis to assess the impact of missing data (44, 45). We used the multiple imputations (MI) approach to address missing data, reported as a number and percent, and re-ran the GEE model for outcomes. The data analyses were performed using SPSS 28.0 (46). Significance was set at alpha = 0.05.

We did not adjust alpha for multiple testing because of the exploratory nature of the analyses (47).

Due to missing data for BMI z-score during the pandemic, mostly due to missing hospital-measured heights to allow accurate calculation of BMI z-score, weight was instead adjusted for in the model and it was not included as an outcome variable.

## Results

**Figure 1:**
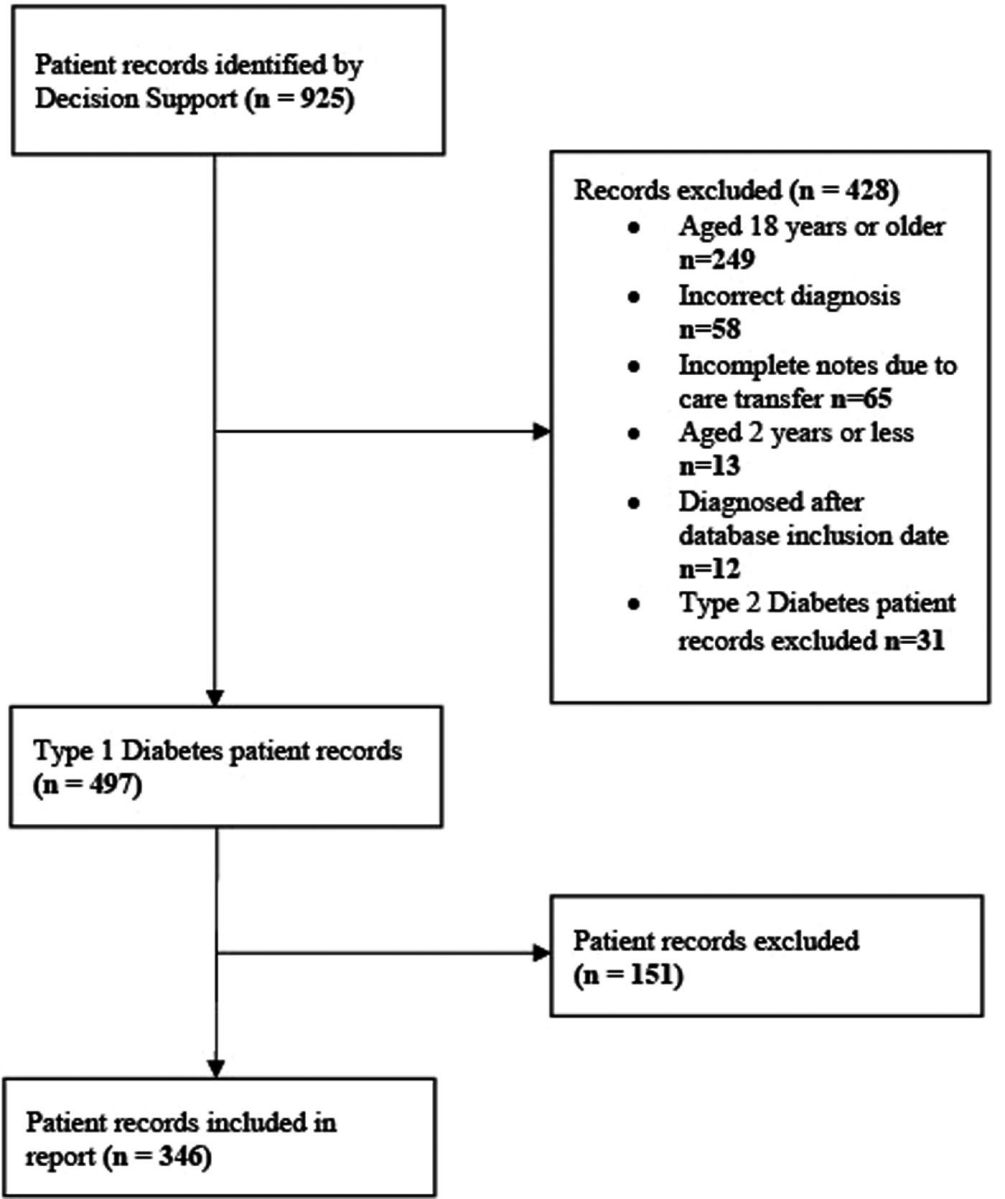
Selection process for included patients.

Participants' characteristics are reported in Table 1. We included 346 patients who met the inclusion criteria (Figure 1). The age at study inclusion was 10.30 ± 3.50 years (range 2.00–16.20 years). Participants had diabetes for 4.50 ± 3.30 years (range 1.00–15.70 years) at inclusion. There were 159 (46.10%) female articipants, and puberty data were not available.

**Table 1:** Characteristics of study participants.

	Pre-pandemic		Pandemic	*P*-value
	2018	2019	2020	
Total Patients, *n*	346	–	–	
Newly Diagnosed, *n*	58	55	81	<0.001*
Sex, *n* (%)				
Male	187 (53.90)	–	–	
Female	159 (46.10)	–	–	
Age at study inclusion, mean (SD), years				
Total	10.30 (3.50)	–	–	
MDI, Male	10.40 (3.70)	–	–	
MDI, Female	10.90 (3.30)	–	–	
Pump, Male	10.10 (3.60)	–	–	
Pump, Female	10.10 (3.30)	–	–	
Diabetes duration, mean (SD), years				
Total	4.50 (3.30)	–	–	
MDI, Male	4.60 (3.90)	–	–	
MDI, Female	4.00 (3.10)	–	–	
Pump, Male	4.50 (3.30)	–	–	
Pump, Female	4.60 (3.20)	–	–	
Treatment, *n* (%)				0.317
MDI	187 (54.00)	142 (41.00)	139 (40.20)	
Pump	159 (46.00)	204 (59.00)	207 (59.80)	
CGM Use, *n* (%)	***n* = 309**	***n* = 331**	***n* = 338**	<0.001
	136 (44.00)	168 (50.80)	205 (60.70)	
Weight, mean (SD), kg	***n* = 333**	***n* = 345**	***n* = 122**	
Total	43.50 (17.50)	48.10 (18.70)	53.28 (18.40)	
MDI, Male	41.20 (18.20)	47.90 (19.20)	56.50 (19.20)	
MDI, Female	41.60 (15.60)	48.00 (18.60)	46.00 (16.40)	
Pump, Male	46.40 (17.20)	48.90 (19.30)	55.10 (18.90)	
Pump, Female	45.00 (18.00)	47.60 (17.50)	52.50 (17.80)	
Weight %ile, mean (SD)	***n* = 333**	***n* = 345**	***n* = 122**	
Total	66.80 (25.70)	69.00 (25.30)	70.40 (25.20)	0.024**
MDI, Male	67.75 (27.41)	66.14 (29.70)	73.80 (28.10)	
MDI, Female	63.83 (24.44)	68.15 (24.00)	57.80 (24.20)	
Pump, Male	67.04 (25.44)	70.10 (24.80)	70.60 (25.50)	
Pump, Female	68.62 (24.17)	71.02 (22.30)	73.20 (21.60)	
Height, mean (SD), cm	***n* = 333**	***n* = 344**	***n* = 110**	
Total	145.70 (21.70)	150.30 (21.20)	155.40 (21.50)	
MDI, Male	142.50 (24.60)	150.50 (23.20)	155.40 (28.50)	
MDI, Female	143.40 (19.80)	147.70 (19.90)	148.90 (18.70)	
Pump, Male	150.90 (20.90)	152.50 (22.70)	160.40 (19.20)	
Pump, Female	147.70 (18.00)	150.00 (17.70)	153.70 (16.50)	
Height %ile, mean (SD)	***n* = 333**	***n* = 344**	***n* = 110**	
Total	59.70 (28.10)	60.90 (28.30)	62.00 (27.80)	0.383**
MDI, Male	58.70 (29.20)	58.00 (29.60)	63.10 (26.40)	
MDI, Female	56.30 (27.80)	55.90 (28.00)	49.30 (30.70)	
Pump, Male	59.70 (27.80)	62.90 (28.90)	64.70 (28.70)	
Pump, Female	65.10 (26.20)	65.30 (25.80)	64.70 (25.70)	
BMI z-score, mean (SD)	***n* = 333**	***n* = 344**	***n* = 110**	
Total	0.70 (1.20)	0.70 (1.00)	0.80 (1.10)	0.004**
MDI, Male	0.80 (1.30)	0.80 (1.10)	1.00 (1.20)	
MDI, Female	0.60 (0.80)	0.80 (0.90)	0.50 (1.00)	
Pump, Male	0.70 (1.40)	0.80 (1.00)	0.80 (1.00)	
Pump, Female	0.60 (1.10)	0.70 (0.90)	0.90 (1.00)	

Total number of participants reported per outcome is bolded and specified by *n*.

SD, standard deviation; MDI, multiple daily injections; *n*, number of participants; CGM, continuous glucose monitor; kg, kilograms; cm, centimeters; BMI, Body Mass Index.

*P*-values compared differences in characteristics pre-pandemic to pandemic phases and were obtained from McNemar's tests unless otherwise indicated.

* *P*-values from Wald *χ*^2^ tests.

** *P*-values from paired *t*-tests.

There was a significant increase in the number of newly diagnosed patients during the first year of the pandemic, when compared to the two years in the pre-pandemic phase (*χ*^2^ = 16.52, *p* < 0.001).

The number of patients using insulin pump therapy increased over time compared to MDI treatment and was stable during the pandemic, as pump start activities shifted to community-based starts with lockdowns (pump use pre-pandemic 2018: 46.00%, 2019: 59.00%; pandemic: 59.80%; *p* = 0.317). In children using MDI treatment, 82.50% had HbA1c measures taken during the pandemic, while 88.90% of children on pumps having pandemic HbA1c data (*χ*^2^ = 2.68, *p* = 0.101). In an exploratory analysis adjusting for age, sex, and diabetes duration, trends for HbA1c did not differ between children using MDI or pump therapy when comparing pre-pandemic and pandemic periods (OR = 0.99; 95% CI 0.94, −1.04, *p* = 0.579). The use of CGM increased over time and climbed further during the pandemic (pre-pandemic 2018: 44.00%, 2019: 50.80%; pandemic: 60.70%; *p* < 0.001).

There was a trend for a rise in BMI z-score during the pandemic when compared to the pre-pandemic phase (*n* = 110, MD = −0.13; 95% CI, −0.22, −0.04, *p* = 0.004) driven by the increase in the weight percentile during the pandemic (*n* = 122, MD = −2.23; 95% CI, −4.16, −0.29, *p* = 0.024). In an exploratory sex-based analysis for BMI z-score, females had a more significant rise in BMI z-score than males during the pandemic (Males *n* = 62, MD = −0.11, *p* = 0.068; Females *n* = 48, MD = −0.16, *p* = 0.023).

There were 216 (63.00%) patients with only 1 HbA1c value during the pandemic. There was no significant change in HbA1c when comparing the pre-pandemic to the pandemic phases (*n* = 343, MD = −0.10; 95% CI, −0.21, 0.01, *p* = 0.076) (Table 2).

**Table 2:** Glycemic outcomes of study participants.

	Pre-pandemic		Pandemic	*P*-value
	2018	2019	2020	
HbA1c, mean (SD) %	***n* = 337**	***n* = 345**	***n* = 343**	
Total	8.10 (1.60)	8.20 (1.50)	8.40 (1.50)	0.076**
MDI, Male	8.40 (1.70)	8.40 (1.80)	8.80 (1.80)	
MDI, Female	8.40 (1.90)	8.60 (1.90)	8.90 (1.80)	
Pump, Male	8.00 (1.20)	8.10 (1.20)	8.10 (1.10)	
Pump, Female	7.70 (1.00)	7.80 (1.00)	8.00 (1.10)	
Hypoglycemia, *n* (%)	***n* = 335**	***n* = 341**	***n* = 341**	
Total	323 (96.40)	332 (97.40)	330 (96.80)	0.819
MDI, Male	68 (21.10)	69 (20.80)	68 (20.60)	
MDI, Female	54 (16.70)	59 (17.80)	59 (17.90)	
Pump, Male	110 (34.10)	111 (33.40)	111 (33.60)	
Pump, Female	91 (28.20)	93 (28.00)	92 (27.90)	
Hyperglycemia, *n* (%)	***n* = 332**	***n* = 338**	***n* = 339**	
Total	278 (83.70)	277 (82.00)	301 (88.80)	0.011
MDI, Male	56 (20.10)	55 (19.90)	59 (19.60)	
MDI, Female	44 (15.80)	45 (16.20)	56 (18.60)	
Pump, Male	95 (34.20)	97 (35.00)	104 (34.60)	
Pump, Female	83 (29.90)	80 (28.90)	82 (27.20)	
DKA				
DKA at diagnosis, *n* (%)	22 (37.90)	23 (41.80)	36 (44.40)	<0.001*
DKA with established diabetes, *n* (%)	19 (5.50)	20 (5.80)	12 (2.90)	<0.001
MDI, Male	5 (26.30)	8 (40.00)	2 (16.70)	
MDI, Female	7 (36.80)	4 (20.00)	4 (33.30)	
Pump, Male	5 (26.30)	6 (30.00)	4 (33.30)	
Pump, Female	2 (10.50)	2 (10.00)	2 (16.70)	
Hospital attendance and hospitalization				
Hospitalization at diagnosis, *n* (%)	51 (87.90)	52 (94.50)	77 (95.10)	<0.001*
Hospitalization with established diabetes, *n* (%)	31 (9.00)	34 (9.80)	26 (7.50)	<0.001
MDI, Male	9 (29.00)	9 (26.50)	4 (15.40)	
MDI, Female	11 (35.50)	8 (23.50)	8 (30.80)	
Pump, Male	7 (22.60)	13 (38.20)	7 (26.90)	
Pump, Female	4 (12.90)	4 (11.80)	7 (26.90)	

Total number of participants with available reported data per outcome is bolded and specified by *n*.

SD, standard deviation; MDI, multiple daily injections; *n*, number of participants; HbA1c, glycosylated hemoglobin A1C; DKA, diabetic ketoacidosis.

*P*-values compared differences in characteristics pre-pandemic to pandemic phases and were obtained from McNemar's tests unless otherwise indicated.

* *P*-values from Wald *χ*^2^ tests.

** *P*-values from paired *t*-tests.

However, more newly diagnosed T1DM patients presented in DKA (*χ*^2^ = 12.94, *p* < 0.001) and more patients required hospital attendance and hospitalization at diagnosis in the unadjusted analysis (*χ*^2^ = 50.94, *p* < 0.001). In contrast, patients with established diabetes had less reported DKA (pre-pandemic 2018: 5.50%%, 2019: 5.80%; pandemic: 2.90%; *p* < 0.001) and hospital attendance and hospitalizations (pre-pandemic 2018: 9.00%, 2019: 9.80%; pandemic: 7.50%; *p* < 0.001) during the pandemic. Hospital attendance and hospitalizations were not significantly different during the pandemic in the GEE analysis (Table 3).

**Table 3:** Changes in diabetes control and diabetes-related morbidities during the COVID-19 pandemic (*n* = 344).

	Effect Estimate		
	Mean difference (95% CI)	Odds Ratio (95% CI)	*p*-value
Main Outcome^a^			
HbA1c	−0.14 (−0.28, 0.01)		0.058
Other Outcomes^b^			
DKA		0.30 (0.12,0.73)	0.009
Hospital attendance and hospitalization		0.57 (0.31,1.04)	0.068
Hypoglycemia		1.11 (0.83,1.49)	0.484
Hyperglycemia		1.38 (1.12,1.71)	0.003

95% CI, 5% Confidence Interval; HbA1c, glycosylated hemoglobin A1c; DKA, diabetic ketoacidosis.

^a^ Analysis was adjusted for age, sex, treatment type (injections vs. pump), sensor use, weight and diabetes-related morbidities (hypoglycemia, hyperglycemia, DKA and hospital attendance and hospitalization).

^b^ Analysis was adjusted for age, sex, treatment type (injections vs. pump) and sensor use.

The occurrence of any hypo- or hyperglycemia, in patients with T1DM was sustained from pre-pandemic levels during the pandemic. A significant number of children had hypoglycemia and this trend continued into the pandemic (pre-pandemic 2018: *n* = 323, 96.40%, 2019: *n* = 332, 97.40%; pandemic: *n* = 330, 96.80%; *p* = 0.819). The hyperglycemic events were noted in the majority of patients in all years of study with a slight rise during the pandemic (pre-pandemic 2018: *n* = 278, 83.70%, 2019: *n* = 277, 82.00%; pandemic 2020: *n* = 301, 88.80%; *p* = 0.011) (Table 2).

To determine if the changes during the pandemic were different when compared to the pre-pandemic phase (2018–2019), we compared the data from the pre-pandemic phase. A significant increase in pump therapy use (2018: 46.00%, 2019: 59.00%; *p* < 0.001), CGM use (2018: 44.00%, 2019: 50.80%; *p* < 0.001), weight percentile (*n* = 333, MD = 1.53; 95% CI, 2.46, 0.59; *p* = 0.001) and BMI z-score (*n* = 333, MD = 0.06; 95% CI, 0.10, 0.02; *p* = 0.002) was found when comparing the data between 2018 and 2019.

The results of the GEE analysis model of the impact of the first year of the COVID-19 pandemic on glycemic control and diabetes-related morbidities are reported in Table 3 (*n* = 344).

During the pandemic, participants with established diabetes maintained their glycemic control (MD −0.14; 95% CI, −0.28,0.01; *p* = 0.058) with significantly less DKA (OR 0.30, 95% CI, 0.12,0.73; *p* = 0.009) and an increase in any hyperglycemic events (OR 1.38, 95% CI, 1.12,1.71; *p* = 0.003) when compared to the pre-pandemic period. There was no change in hospital attendance and hospitalizations (OR 0.57, 95% CI, 0.31,1.04; *p* = 0.068) or hypoglycemic events (OR 1.11, 95% CI, 0.83,1.49; *p* = 0.484).

To address the effect of missing data during the pandemic with virtual visits, we conducted multiple imputations and repeated the GEE model analyses which confirmed the results, and that the missingness of data did not impact the analysis conclusions (Table 4).

**Table 4:** Sensitivity analysis using multiple imputations and generalized estimating equation model to assess glycemic outcomes (*n* = 346).

	Effect Estimate		
	Mean difference (95% CI)	Odds Ratio (95% CI)	*p*-value
Main Outcome^a^			
HbA1c	−0.09 (−0.30,0.12)		0.404
Other Outcomes^b^			
DKA		0.31 (0.12,0.79)	0.014
Hospital attendance and hospitalization		0.58 (0.32,1.07)	0.081
Hypoglycemia		1.06 (0.80,1.40)	0.707
Hyperglycemia		1.26 (1.01,1.58)	0.042

95% CI, 95% Confidence Interval; HbA1c, glycosylated hemoglobin A1c; DKA, diabetic ketoacidosis.

^a^ Analysis was adjusted for age, sex, treatment type (injections vs. pump), sensor use, weight and diabetes-related morbidities (hypoglycemia, hyperglycemia, DKA and hospital attendance and hospitalization).

^b^ Analysis was adjusted for age, sex, treatment type (injections vs. pump) and sensor use.

## Discussion

The COVID-19 pandemic fundamentally shifted models of care delivery for children living with T1DM. The short-term outcomes of patients living with T1DM during the pandemic continue to unravel, and it will be many years before the full impact of the pandemic on diabetes outcomes is fully understood.

This retrospective study compared data from the two years pre-pandemic to the first year of the pandemic in children living with T1DM. When assessing glycemic control, the HbA1c remained stable during the first year of the pandemic. This conclusion, while contrary to our hypothesis, is consistent with some of the emerging evidence for stability of pandemic time glycemic control trends (20, 48–53), and some studies even reported an improvement in glycemic control (22, 54). The stability or potential improvement in glycemic control during the pandemic may be related to enhanced awareness of children and caregivers during lockdowns with more attention paid to glycemic trends, increased patient-caregiver collaboration with managing glycemia, and the development of predictable management routines (22, 54). Importantly, the data on stability of glycemic trends in T1DM were reported from upper-middle or high-income countries with infrastructure to conduct virtual visits and sustained access to glucose monitoring supplies and treatments (20, 22, 48, 49, 51, 52, 54). A study from India based on reported self-monitoring of glucose data reported a deterioration in glycemic control during lockdowns due to the reduced availability of insulin and glucose test strips in the early stages of the pandemic (55). For virtual and hybrid models of diabetes care to be universally effective and sustainable, the disparity in access to diabetes healthcare professionals, supplies, and digital resources need to be eliminated.

The number of patients presenting with new onset T1DM rose during the pandemic, and more patients presented in DKA when compared to the pre-pandemic phase. In contrast, fewer patients with established T1DM diagnosis had DKA during the pandemic phase. While the reported number of cases for these outcomes were relatively small, the data are consistent with current evidence reporting an increase in DKA and its severity at diagnosis (21, 26–29, 56–58). These findings are likely related to delays in seeking care due to concerns about the increased risk in contracting the COVID-19 virus in healthcare facilities, and caregivers abiding by health systems messaging to divert care away from hospitals to prioritize resource utilization for COVID-19 activities (26). Public health education campaigns may be helpful in this phase of the pandemic to remind caregivers of the symptoms of diabetes and DKA to allow early diabetes diagnosis and mitigate the risk and severity of DKA.

Some studies reported an increase in DKA rates in children with established diabetes during the first lockdown within the first five months of the pandemic, with a return to or below pre-pandemic levels within the first few months (48, 56).

Consistent with our results, there is evidence to suggest that the pandemic was not associated with increased hospital attendance and hospitalizations in those with established diabetes (20). The rapid adoption of telemedicine and diabetes team accessibility, the pre-existing high levels of access to technologies including CGM and pump therapy, and increased attention to glycemic trends likely helped limit hospital attendance and hospitalizations during the pandemic (20–22, 24, 59, 60).

The pandemic was associated with higher rates of hyperglycemia when compared to the pre-pandemic phase despite stable glycemic control. It is possible that reported hyperglycemia is related to enhanced attention to glucose profiles while in lockdown, the use of CGM, and alterations in dietary patterns and physical activity levels, with children being less active and using technology for longer periods than in pre-pandemic times (22, 54, 61–63). Another explanation is that while the reporting of hyperglycemia was based on the occurrence of these events, the duration of time when the patient had a normal glucose likely contributed to stable glycemic control.

The burden of hypoglycemia remained high throughout the study period. Hypoglycemia is a frequent accompaniment of T1DM (64–67). For patients living with T1DM and their families, glycemic variability and avoiding hypo- and hyperglycemia are a consistent and substantial burden in diabetes management (64–69).

Recent reports suggest that glucose sensor data of time in range (TIR), hypoglycemia, and hyperglycemia were stable or improved early in the pandemic; the trends reverted when patients returned to school and other activities with the relaxation of social distancing measures (23, 49, 51–54). There is a need to assess approaches to maintain glycemic trends by limiting glycemic excursions in T1DM.

Our data suggest that the weight percentile and BMI z-score increased during lockdowns and this trend continued from the pre-pandemic phase. The possible explanations for the pandemic results include the impact of age and growth, puberty, diabetes duration, anabolic effects of insulin over time on body mass, and children having lower physical activity, sedentary behaviours, and changes in dietary habits during lockdown when compared to the pre-pandemic phase (48–51, 70–74). Further assessment of the factors contributing to this upward body mass trend in children with diabetes is warranted.

We noted a significant increase in the use of glucose monitoring technology including CGM when comparing the pre-pandemic years of 2018 and 2019. As CGM use was also noted to increase during the pandemic year, it is likely that this trend was not because of the pandemic alone. Rather, patients were already transiting to use technology and likely would have continued to adopt these tools even without the pandemic.

### Strengths & limitations

This study compared glycemic trends and diabetes-related outcomes before and after the start of the pandemic. The longitudinal data available for a relatively large sample size and using the GEE approach allowed data comparison over time.

There were some limitations to the study. This is a single center retrospective study from a high-income country, which may limit generalizability. In addition, the confounder of time may lead to some measures changing over time regardless of the pandemic. The HbA1c data were also limited with some patients only having one or two HbA1c values during the pandemic, when compared to four annual tests in the pre-pandemic phase due to disruption of point-of-care clinic and laboratory-based services. This limitation required a pragmatic decision to use existing data. The disruption of children's access to laboratories and point-of-care testing facilities was significant during the early part of the pandemic, and the data available reflected this limitation.

## Conclusions

In patients with T1DM, glycemic control was comparable during the first year of the COVID-19 pandemic to the pre-pandemic phase. The number of new patients with T1DM and those presenting in DKA at diagnosis increased during the pandemic, while those with established diabetes saw a decline in DKA. The burden of hypo- and hyperglycemia was substantial throughout the study period.

There is a need for healthcare systems to equip children and families living with T1DM with the technological and digital infrastructure necessary to support diabetes care and maintain glycemic outcomes and lower the risk of co-morbidities and complications. These approaches to care require a commitment to equity in access of all children to maintain and improve diabetes control and outcomes globally.

## Data Availability

The raw data supporting the conclusions of this article will be made available by the authors, upon reasonable justification.
